# Challenges and opportunities in the development and implementation of zero leprosy roadmaps in low-endemic settings: Experiences from Bolivia, Pakistan, and Togo

**DOI:** 10.1371/journal.pntd.0013009

**Published:** 2025-04-28

**Authors:** Anil Fastenau, Felicitas Schwermann, Abundio Baptista Mora, Issaka Maman, Thomas Hambridge, Matthew Willis, Sundeep Chaitanya Vedithi, Nimer Ortuño-Gutiérrez

**Affiliations:** 1 Marie Adelaide Leprosy Center (MALC), Karachi, Pakistan; 2 German Leprosy and Tuberculosis Relief Association (GLRA), Wuerzburg, Germany; 3 Department of Health, Ethics & Society, Faculty of Health, Medicine and Life Sciences, Maastricht University, Maastricht, The Netherlands; 4 Department of Global Health, Institute of Public Health and Nursing Research, University of Bremen, Bremen, Germany; 5 Heidelberg Institute of Global Health (HIGH), University of Heidelberg, Heidelberg, Germany; 6 Damien Foundation, Santa Cruz, Bolivia; 7 Institut National d’Hygiène, Lomé, Togo; 8 Erasmus MC, University Medical Centre Rotterdam, Rotterdam, The Netherlands; 9 Department of Medicine, University of Cambridge, Cambridge, United Kingdom of Great Britain and Northern Ireland; 10 American Leprosy Missions, Greenville, South Carolina, United States of America; 11 Damien Foundation, Brussels, Belgium; Shandong Provincial Institute of Dermatology and Venereology, CHINA

## 1. Background

**Fig 1:**
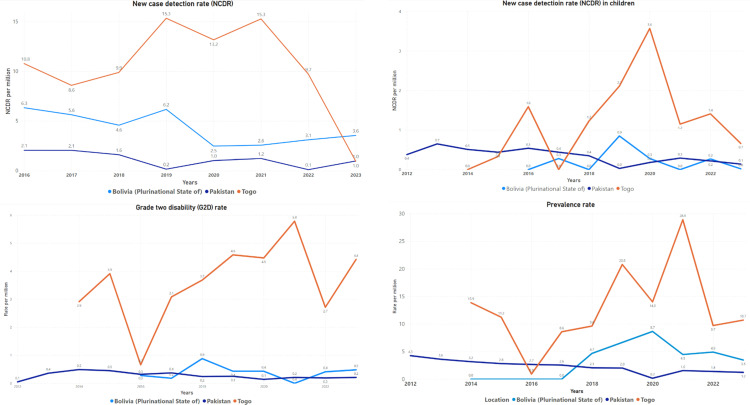
Key indicators of leprosy in Bolivia, Pakistan, and Togo.

Leprosy, caused by *Mycobacterium leprae*, remains a significant global health challenge with up to 200,000 new cases detected annually [1]. The World Health Organization (WHO) targets interrupting transmission with no autochthonous new cases in 120 countries by 2030 [1]. To this end, WHO and the Global Partnership for Zero Leprosy have developed a toolkit for implementing country-owned “Zero Leprosy” roadmaps [2]. In Bolivia, Pakistan, and Togo, which are considered low-endemic with new case detection rates per million ranging from 1 in Togo and Bolivia and 3.6 in Pakistan in 2023 (Fig 1) [3], the development of zero leprosy roadmaps has revealed a mix of unique and shared challenges. In Bolivia, Pakistan, and Togo, transmission is declining as illustrated by new case detection rate per million in children ≤14 years old in 2023 that varies from zero in Bolivia, 0.1 in Pakistan, to 0.7 in Togo (Fig 1). However, this rate varies from the years prior to 2023 as for instance in 2022 all three countries notified children among new leprosy cases illustrating that transmission is persistent.

We synthesize insights from recent experiences in developing zero leprosy roadmaps in these countries supported by international non-governmental organizations as German Leprosy and Tuberculosis Relief Association (GLRA) and Damien Foundation from Belgium, highlighting barriers and opportunities (Table 1).

**Table 1:** Challenges and opportunities for the development and implementation of zero leprosy roadmaps in low endemic settings.

Challenges	Opportunities	Examples of successful approaches implemented
Limited clinical expertise of health staff:		
• Poor clinical skills for proper diagnosis and treatment of leprosy • Limited inclusion of histopathology and slit-skin smears for diagnosis • Absence of molecular diagnostics and confirmation tests for accurate leprosy diagnosis • Primary healthcare workers not familiar with leprosy • Reduction of curricula for pre- and post-graduate training on leprosy • Limited budget for clinical training and refresher training	• World Health Organization (WHO) online open courses can be complemented with practical training sessions • Apps using artificial intelligence might be used for triage and referral for confirmation from primary health staff • Integration of training with skin neglected tropical diseases (Skin-NTDs) • Building a referral system for confirmation including new q-PCR diagnostic tests	• Restoring slit-skin smears (SSS) under external quality assessment (EQA) • Translating experiences from research using q-PCR for the confirmation of leprosy and targeted sequencing for assessing resistance, and Whole Genomic Sequencing for assessing molecular transmission in Comoros [8]
Lack of reliable clinical and epidemiological data:		
• Only aggregated data is available • Poor recording and reporting • No quality assurance of data notified • Health staff not trained in data reporting and analysis • Limited clinical expertise contributing to gaps in reliable leprosy data • No GIS data at household and community level	• New tools available for digital data collection and analysis • Possibility of including a tracker module to the health information system in place, i.e., DHIS-2	• Global Leprosy Mapping Initiative by ILEP piloting GIS mapping in Bolivia, Nepal, and Senegal [21]
Lack of systematic contact examination:		
• Limited active case-finding activities • Absence of retrospective contact tracing • Lack of indicators and data for assessing coverage and yield of contact tracing • Limited countrywide implementation of single-dose rifampicin as post-exposure prophylaxis (SDR-PEP)	• Integrating SDR-PEP in contact tracing invigorates the national leprosy programs • LEMT useful for targeting additional contact examination in phase 1 • New strengthened PEP regimens are under investigation	• Systematic contact tracing and SDR-PEP substantially decreased leprosy in Morocco a low endemic setting for leprosy [10] • Single-dose rifapentine has stronger protection compared to SDR [19] and has been studied in low-endemic settings
Poor implementation of SDR-PEP:		
• Lack of resources for training health staff and purchasing rifampicin • Poor geographical access to remote and hard-to-reach rural settings • Not reliable data for retrospective contact investigation and provision of SDR-PEP • Lack of understanding of the rationale of SDR-PEP among stakeholders and decision-makers	• WHO guidelines for SDR-PEP • SOPs developed by GPZL for facilitating implementation • Integration of SDR-PEP with other outreach activities	• Successful implementation of SDR-PEP in Morocco[10] and Bolivia [5]
Weak data management and surveillance systems:		
• Fragmentation of health information systems • Paper-based data collection • Lack of quality assurance of data notified • Lack of analysis of data collected	• LEMT developed by WHO motivates leprosy programs to reinforce the recording, reporting, and surveillance of leprosy • DHIS-2 widely implemented using aggregated data	• Successful retrospective mapping and surveillance of leprosy patients in Cambodia [13]
Stigma and discrimination: • High levels of stigma and discrimination among people affected by leprosy and the community • Lack of community engagement in low endemic settings, i.e., in the majority of countries there is an absence of associations of people affected by leprosy	• Integration of people affected by leprosy in the process of designing, implementing, and assessing health interventions • Strengthening of associations of persons affected by leprosy and other NTDs	• Successful implementation of community-based approach in Bolivia [5]

## 2. Challenges in leprosy elimination in low-endemic settings

### 2.1. Delayed diagnosis and limited clinical expertise

Confirmatory diagnosis of leprosy, particularly in low-endemic settings, poses a significant challenge. In primary healthcare settings, diagnostic delays in leprosy can be attributed to lack of knowledge of cardinal clinical signs among healthcare workers (which include three cardinal sings: (1) definite loss of sensation in a skin lesion, (2) thickened or enlarged peripheral nerve with loss of sensation and/or weakness of the muscles supplied by that nerve, and (3) microscopy detection of bacilli in a slit-skin smear) [4]. Also, lack of knowledge among communities and individuals is a factor influencing delay of diagnosis [5]. In Bolivia and Togo, low-endemicity has led to a diminished focus on leprosy. As a result, healthcare providers fail early diagnosis which increases the risk of transmission of leprosy in the community and diagnosis of leprosy with permanent disability. While there are molecular tools available for confirmation of cases of high bacterial loads [6] these tools are not yet widely recommended or implemented and less sensitive tests such as histopathology and slit-skin smears are used. Hence, the lack of capacity for early clinical diagnosis and non-inclusion of molecular diagnostic tests for confirmation pose a major challenge for leprosy elimination in low-endemic settings.

### 2.2. Data availability and reliability

In low-endemic settings, a significant challenge in leprosy elimination is the limited availability of granular and reliable epidemiological data. As case numbers decline, routine surveillance systems often weaken, leading to underreporting, inconsistent data collection, and absence of quality assurance programs. In Togo and Bolivia, quality of data is compromised by inadequate diagnostic and treatment capabilities, poor record-keeping, and fragmented health information systems. Moreover, social stigma can discourage individuals from seeking care, resulting in undiagnosed cases and underreporting. Notably, the available data is mostly aggregated and collected at the district level. For targeted and (cost-)effective active detection, however, granular detail is needed, including GPS coordinates at community or household level while ensuring confidentiality [7].

### 2.3. Lack of systematic contact examination and effective active case detection

The most critical bottleneck in interrupting transmission is under detection and delay of diagnosis, impacting patients and their household contacts who have higher risk of becoming infected [8,9]. Systematic screening of household contacts of index cases conducted by well-trained staff is therefore essential for leprosy elimination, especially in combination with post-exposure prophylaxis (PEP) with a single dose of rifampicin (SDR) that significantly reduces leprosy at the population level [10]. However, in many low-endemic countries, including Bolivia, Pakistan, and Togo, leprosy is not a priority for governments. As a result, all the household and social contacts of people affected by leprosy are not routinely examined nor monitored, resulting in continued transmission, and disabilities due to delayed diagnosis.

Even in high endemic settings leprosy is clustered in geographic areas, therefore there is a need to tailor the active case finding. For instance, in Comoros a door-to-door screening of entire villages population based on the historical data of new leprosy cases detected 5 years ago unmasked an important number of leprosy cases [11]. Also, in India Leprosy Case Detection Campaigns where accredited social health activists visited door-to-door endemic villages selected by the historical detection of leprosy yield similar results [12].

### 2.4. Logistical and infrastructural barriers to implementation of post-exposure prophylaxis (PEP)

Low-endemic countries face severe logistical and infrastructural challenges. In Togo, Bolivia, and Pakistan, access to leprosy healthcare services is rare in remote rural areas which implies high costs for accessing health facilities. The lack of healthcare personnel and diagnostic tools exacerbates this problem. In addition, a lack of cost-effectiveness analysis of, e.g., skin camps in low-endemic areas might result in inefficient distribution of scarce resources. Ultimately, the financial resources needed to increase countrywide coverage for contact examination and SDR-PEP are insufficient. Furthermore, implementation of systematic retrospective contact identification (contacts of new leprosy cases diagnosed in the past 5–10 years) and tracing in low-endemic areas remains also difficult.

### 2.5. Data management and monitoring systems

Effective epidemiological data management is essential for leprosy case-holding and monitoring progress toward elimination. However, data systems in low-endemic settings are often fragmented. The WHO recommends adopting electronic case-based data systems such as DHIS2, however, uptake of this is limited and a “tracker DHIS2-based system” has not yet been made available. Mapping tools for disease surveillance and progress toward elimination, such as the Leprosy Elimination Monitoring Tool (LEMT), have been introduced in several countries, but there is a need for more granular data to track transmission hotspots and improve intervention targeting. Furthermore, retrospective active case finding and Geographic Information System-Mapping (GIS-Mapping) of historical leprosy cases remains a major challenge, though it has been successful in low-endemic settings like Cambodia [13]. Bolivia, Pakistan, and Togo have all identified gaps (lack of reliable historical database, lack of digital tools for data collection including GPS coordinates, lack of integration of surveillance in the national health information system, etc.) in their surveillance and data management systems. In Pakistan, a new tracker DHIS2-based system is being developed to better monitor cases and interventions, but integrating and managing data from remote hard-to-reach areas remains a challenge.

### 2.6. Social stigma and community engagement

Leprosy continues to carry a heavy social stigma, which hinders both diagnosis and treatment. Individuals affected by leprosy may experience delays in seeking medical help due to fear of discrimination, leading to worsened disease outcomes and further transmission. This stigma is particularly prevalent in rural areas, as seen in Bolivia [5]. Community engagement and public health campaigns are critical for reducing stigma and encouraging early treatment. In these countries, efforts to involve individuals affected by leprosy in awareness campaigns have shown progress but need to be scaled up.

### 2.7. Management of leprosy patients and leprosy related complications

Although case-fatality is low, people affected by leprosy might develop leprosy immunological reactions before diagnosis, during treatment, and after release from treatment [4]. Again, this needs high clinical skills for prevention, recognition, and management, otherwise long-life disabilities are provoked [14]. It is estimated that around four million of people suffering from disability related from leprosy [1]. In Togo, despite the reduction of incidence the disability increased [15], which might be related to poor geographical access for management, lack of clinical skills among healthcare workers, or overestimation. Also, length of treatment which is up to 12 months for multibacillary cases might be a barrier for compliance, as well as the dapsone-related hypersensitivity leading to severe adverse events that is part of the multidrug-therapy besides rifampicin and clofazimine for the treatment of both paucibacillary and multibacillary forms of leprosy [4]. The length of treatment and its safety might be reduced with the addition of new drugs as bedaquiline that are proven safe and with long half-life [16].

## 3. Opportunities and strategic interventions for the implementation of zero leprosy roadmaps

### 3.1. Enhanced surveillance and active case finding

A key component of Zero Leprosy Roadmaps includes implementation of robust surveillance systems integrated into the national health information system. In low-endemic settings, active case-finding strategies need to be carefully designed. In Pakistan, GIS-Mapping based on the validated database from more than two decades has been used effectively to identify transmission hotspots in Karachi, and similar strategies could be employed in Bolivia and Togo where this strategy is not implemented yet because of lack of reliable historical data. Implementing integrated approaches, such as combining leprosy screening with other skin-Neglected Tropical Diseases (NTDs) [17], has the potential to improve case detection rates. We propose that systematic contact tracing and examination of all the leprosy cases of the last 10 years in Bolivia, Pakistan, and Togo could play a vital role in interruption of transmission. Furthermore, such intensive contact examination should be combined with the implementation of PEP with single dose rifampicin to achieve zero leprosy which is systematically implemented in Pakistan, rather than as a pilot study in Bolivia [5] and Togo [18] where national policy needs to be based on local studies addressing safety and challenges of operational implementation. In a low endemic setting for leprosy in China, rifapentine showed stronger protection (84%) compared to SDR [19] and could be implemented to maximize impact of contact tracing and active case detection for zero leprosy.

### 3.2. Strengthening diagnostics and capacity building

The development of diagnostic tools, including molecular diagnostics and serological tests for subclinical leprosy, offers hope for earlier and more reliable detection of *Mycobacterium leprae* infection. Therefore, improving diagnostic capabilities is essential in low-endemic settings like Bolivia, Pakistan, and Togo where slit-skin smears and pathology are used for confirmation. However, for these diagnostic tools to be effective, there needs to be a concerted effort to build the capacity of healthcare workers to properly use diagnostic tests. In Bolivia, Pakistan, and Togo, training and educating healthcare personnel to recognize leprosy symptoms and applying lab-based PCR diagnostic tests is crucial for confirmatory diagnosis, surveillance of antimicrobial resistance, and assessing pathogen transmission using genomics surveillance. Furthermore, telemedicine can be strengthened in addition to capacity building for early presumptive cases and referrals for confirmation as well as strengthening laboratory networks to improve diagnostic accuracy. Artificial intelligence apps can be used in primary healthcare as a triage for referral combined with physical examination[20].

### 3.3. Integrating leprosy with other NTDs

Given the low incidence of leprosy in these countries, integrating leprosy services with other skin-related NTDs, such as cutaneous leishmaniasis, Buruli Ulcer, or Yaws, can optimize resource utilization and ensure sustainability [17]. Togo has already successfully started the integration of leprosy with other NTDs, since 2018 the leprosy program is integrated in the National Neglected Tropical Diseases Program [15], and Bolivia and Pakistan could benefit from similar integrated approaches. An integrated protocol for molecular diagnosis and testing the seroprevalence of multiple NTDs is one possible solution.

### 3.4. Reducing stigma and involving communities

Community engagement is essential to overcoming the barriers posed by stigma. In Togo, Pakistan, and Bolivia [5], efforts have been made to involve persons affected by leprosy in decision-making processes, which has helped to shift community perceptions. Educating by public health campaigns aimed at reducing stigma in health services and promoting early treatment are crucial in all three countries.

### 3.5. Improving epidemiological data management and monitoring

The WHO recommends that countries implement electronic case-based data systems using DHIS2 modules to improve case tracking and data management of leprosy and other NTDs [1]. Expanding the use of these systems and strengthening their integration within health information systems will help to monitor their progress toward elimination more effectively. Moreover, developing communication strategies in low-endemic settings clarifying milestones and goals of the Zero Leprosy Roadmap will help manage public expectations and sustain momentum.

## 4. Conclusion

The development and implementation of zero leprosy roadmaps in low-endemic settings, such as Bolivia, Pakistan, and Togo, present significant challenges to achieving the 2030 targets. Key barriers include the lack of reliable data, systematic contact examination, and clinical expertise, as well as delays in diagnosis, logistical constraints, and the pervasive social stigma surrounding the disease. However, these countries have also demonstrated opportunities for innovation and integration within their leprosy elimination strategies. By fostering country ownership and community engagement, leveraging advanced diagnostics, digitizing transmission surveillance, strengthening epidemiological data monitoring, and integrating NTD services, they are setting the foundation for transformative progress.

The lessons learned from Bolivia, Pakistan, and Togo could be a game changer for leprosy elimination in other low-endemic countries. These approaches not only accelerate the path toward eliminating this ancient disease within these nations but also have global implications, offering a scalable blueprint to combat leprosy and achieve elimination targets worldwide.
